# Prescription Department

**Published:** 1895-09

**Authors:** 


STOMATITIS.

In the herpetic form, Dr. Marfan speaks favorably of a mixture thus composed:

℞. Aquæ dest............... ℨiiss.

Glycerini............... ℨiiss.

Iodini..................gr. vi.

Potassii iodidi............gr. vi.

M.—Sig.: For local use. Apply to lesions.—*La Medecine Moderne.*

FOR DIARRHŒA.

By Dr. J. W. McGlaughlin.

℞. Pulv. catechu ..........gr. xii.
Pulv. opium.............gr. xii.
Tannic acid.............gr. xii.
Acetate lead............ gr. xii.

M.—Sig.: Chart xxiv; for children, one every two hours, or as is needed. (Adults chart xii.)

FOR GASTRODYNIA.

By Dr. Abercrombie.

℞. Ferri sulph..............gr. xx.
Pulv. aloes...............gr. x.
Pulv. cinnamoni co......ℨj.

M. et ft. pil. No. xx.
Sig.: One after each meal.

BRONCHIAL CATARRH OF WHOOPING COUGH.

By Prof. Bartholow, Philadelphia.

℞. Tinct. aconiti rad.......m. xvi.
Tinct. opii. deodorat....m. viij.
Syrupi tolutani.........ℨvij.
Syrupi scillæ comp.
Aquæ lauro-ceras, ......aa ℥j.

M.—Sig.: Teaspoonful every two, three, or four hours. It is an excellent cough mixture, under any circumstances.

FOR CYSTITIS.

By Prof. A. J. C. Skene.

℞. Acidi benzoici.

Sodii borat.............aa ʒ ij.

Infus. buchu............℥ xij.

M.—Sig.: Wineglassful three or four times a day.

LUMBAGO.

By Dr. Clarence G. Hellister.

℞. Potass. iodidi...........℥ ss.

Potas. bromidi......... ℥ ss.

Tr. colchici sem.

Syr. aurantii cort......f. ℥ jss.

Aquæ q. s. ad..........f. ℥ vj.

M.—Sig.: One teaspoonful three times a day, or increased until the bowels are slightly acted upon.

RHEUMATISM AND SPRAIN LINIMENT.

By John J. Gage, M. D.

℞. Gum camphor..........℥ ss.

Chloroform..... ......℥ ss.

Tr. aconiti.. ..........ʒ j.

Tr. arnica.... .........ʒ ij.

Tr. opii................ʒ ij.

Oil wintergreen........ʒ ss.

Soap liniment, q. s., ad. ℥ iv.

M.—Sig.: Apply as often as necessary, and cover with flannel or oil silk.

PRURITUS OF THE VULVA.

By Dr. Oliver, London.

℞. Cocaine................ gr. x.
Chloral..................gr. xij.
Glycerin..... ..........℥j.

Sig.: To be applied three or four times daily.

